# Formulation of New Chewable Oral Dosage Forms of Meclizine and Pyridoxine Hydrochloride

**DOI:** 10.1155/2023/5512379

**Published:** 2023-07-29

**Authors:** Wafa M. Al-Madhagi, Arwa Alshargabi, Abdulkarim K. Y. Alzomor, Olla Sharhan

**Affiliations:** ^1^Department of Pharmaceutical Chemistry, Faculty of Pharmacy, Sana'a University, Sana'a, Yemen; ^2^Department of Pharmacy, Faculty of Medicine and Health Sciences, Al Nasser University, Sana'a, Yemen; ^3^Department of Pharmacy, Faculty of Medical Sciences, Saba University, Sana'a, Yemen; ^4^Department of Pharmacy, Department of Chemistry, Faculty of Sciences, Faculty of Medicine and Health Sciences, Thamar, Yemen

## Abstract

Nausea and vomiting are symptoms associated with a lot of diseases and oral tablets may be unprofitable for patients especially those suffering from nausea and vomiting. Therefore, this study aimed to formulate a new meclizine and pyridoxine combination formula for chewable tablets and provide rapid drug absorption and decrease motion sickness. The new chewable formulation has been prepared to provide fast action, is more acceptable, and could be used for all age categories. Seven trials haves been carried out to prepare to find the suitable one where formula 7 of the chewable gum preparation exhibited good taste and hardness, while the gelatin formulation give an accepted formula after four trials with better taste and good acceptance. The prepared formulations give a dissolution profile of meclizine (95.53–102.8%) and pyridoxine (99.25 ± 115%) and assay (98 + 0.05–99.3 ± 0.8%) for meclizine and (97 ± 0.9–100.0 ± 0.08%) for the pyridoxine in three prepared formulations of chewable tablets. Followed by the evaluation, the formulation and testing them on human volunteers are carried out to confirm their effect to ensure acceptance and fast actions. The finding is promising for preparing a new route of administration of meclizine and pyridoxine combination to be used in the market.

## 1. Introduction

Formulation of oral dosage forms is suitable for various drugs, but they are challenging to formulate if the active substances have poor dissolution rates or low bioavailability. Therefore, different techniques have been used to overcome this problem and enhance the dissolution rate and solubility of the drug [1–4]. One of these techniques is the formulation in the buccal cavity that would have great intensity because of the high blood supply that would increase absorption. As an example, meclizine hydrochloride has poor water solubility [5–7], and this poor solubility leads to a slow rate of oral route absorption. Therefore, enhancing the drug dissolution would help ensure its maximum therapeutic utility [1, 8–10]. Meclizine hydrochloride (MCZ) is a first-generation antihistamine of the piperazine class drug that is used in the motion sickness (H1 receptor antagonist) treatment and used alone or in combination with pyridoxine. Meclizine and pyridoxine formulation are drugs of choice in pregnancy and for motion sickness relief. Moreover, meclizine and pyridoxine have been used to eliminate psychomotor and cognitive impairment activity [1]. Pyridoxine which is called vitamin B6 can be used for the prevention of the effect of peripheral neuropathy and used in combination with isoniazid for tuberculosis treatment. In addition, pyridoxine has proven its activity in the treatment of pyridoxine-dependent epilepsy. In this research, meclizine and pyridoxine would be formulated as chewable tablets to enhance and fasten their absorption.

Chewable tablets are mostly chewed in the mouth before swallowing. The main aim of the chewable tablet is to give a proper unit dosage form of medication that can easily be given to children or to the elderly who have difficulty swallowing a tablet intact [11, 12]. Chewable tablets are taken to disintegrate smoothly in the mouth at a moderate rate either with or without actual chewing; the properties of chewable tablets have a smooth texture upon disintegration, are pleasant tasting, and leave no bitter or unpleasant taste. Geriatric and pediatric patients and traveling patients who may not have ready access to water are most in need of easy-swallowing dosage forms like chewable tablets. One or more approaches are followed to arrive at a combination of the formula and the process that result in a product with well-organoleptic properties. Such a substance must have acceptable flow, compressibility, and stability properties [11, 12]. Many pregnant women and children do not prefer swallowing tablets based on an initial survey run by this study where 46% of the respondents are suffering from motion sickness and 39.3% are pregnant women and prefer to have a chewable formulation than the oral swallowed tablet. Moreover, chewing may help the traveler to forget about nausea and the possibility of increasing the absorption because of a high number in the blood vessels in the buccal cavity. Therefore, the present study was designed to prepare a new formulation of chewable formulation of meclizine and pyridoxine combination that may enhance their absorption and increase patient acceptance as antiemetic has been carried out.

## 2. Materials and Methods

### 2.1. Survey

To estimate the preferable dosage form of meclizine and pyridoxin combination for nausea and vomiting treatment, an initial survey was conducted following the descriptive analysis of data, and the target population *was* women (pregnancy and nonpregnancy) and the general community. *Studying sample*: random. *Survey method*: questionnaire. *Studying Society*: 153 cases.

 
*Sample size calculation for study*: https://www.rasoft.com/samplesize.html
 
*Inclusion*: pregnancy, children, and people traveling with motion sickness
 
*Exclusion*: people have not suffered from motion sickness; women have not afforded it previously

### 2.2. Formulation

#### 2.2.1. Materials of Chewable Gum Tablet (Chewing Gum)

The materials used for preparation of chewable gum tablets were as follows: meclizine HCL (active ingredient, Selectchemie AG), pyridoxine HCL (active ingredient, Shanghai OSD), saccharine (sweetening agent), citric acid (flavoring agent), gum base, glycerol (humectant), and peppermint powder (flavoring agent, Aromsa).

#### 2.2.2. Materials of Chewing Tablet (Mentos-Like Tablet)

The materials used for preparation of chewable tablets were as follows: meclizine HCL (active ingredient, Selectchemie AG), pyridoxine HCL (active ingredient, Shanghai OSD), saccharine (sweetening agent), citric acid (flavoring agent), gum base, xanthan gum (viscous agent, DLS pharma), microcrystalline cellulose 101 (MCC) (binder, FMC), glycerol (humectant), and peppermint powder (flavoring agent, Aromsa).

#### 2.2.3. Materials of Gummy Tablet

The materials used for preparation of gummy tablets were as follows: meclizine HCL (active ingredient, Selectchemie AG), pyridoxine HCL (active ingredient, Shanghai OSD), gelatin (base), saccharine (sweetening agent), citric acid (flavoring agent/souring), and methylparaben (preservative).

### 2.3. Methods

#### 2.3.1. Procedure for Formulation

A chewable tablet is prepared in three forms to be suitable and acceptable in different ages, which are gum-like, Mentos-like, and gummy-like as follows.

#### 2.3.2. Chewable Tablet (Gum-Like) Formulation

The formulation was carried out by the direct compression method using various ratios of the directly compressible gum base. The excipients used were saccharine (sweeting agent), citric acid (flavor), and gum base glycerol (humectant). We grind saccharin and citric acid together until they become smooth, mix meclizine and pyridoxine with saccharine and citric acid, and add flavor and food dye which has been preground to be homogeneous, after mixing all ingredients together to form homogeneous gum base. Kneading lasts 15 minutes until it becomes homogeneous with all its components. After that, the prepared formula is then compressed into a tablet.

#### 2.3.3. Procedure for Formulation of the Chewable Tablet (Mentos-Like Tablet)

The excipients used were saccharine (sweeting agent), citric acid (flavoring agent), gum base, xanthan gum (viscous agent), microcrystalline cellulose 101 (MCC) (binder), glycerol (humectant), and peppermint powder (flavoring agent). We grind saccharin and citric acid together until they becomes smooth, mix meclizine and pyridoxine with saccharine, citric acid, microcrystalline cellulose 101 (MCC), and xanthan gum (viscous agent), and add flavor and food dye which has been preground to be homogeneous, after mixing all ingredients together to form homogeneous gum base. Kneading lasts 15 minutes until it becomes homogeneous with all its components. Then, the prepared formula is then compressed into a tablet.

#### 2.3.4. Procedure for Formulation of Gummy Tablet

The excipients used were gelatin (base), saccharin (sweeting agent), citric acid (flavoring agent/souring), and methylparaben (preservative).

We take 1 g of gelatin and add citric acid, meclizine, pyridoxine, sorbitol, saccharin, and methylparaben with 10 ml water. Then, we mixed all together, then added color and flavor, and poured to a mold. The gelatin tablets are weighed separately for each one gram; after weighing gelatin tablets for each one gram separately, they are collected in plastic boxes in the presence of silica gel, which prevents moisture.

### 2.4. Quality Control Tests

#### 2.4.1. Disintegration

Water was selected as media for the disintegration as the buccal pH is between 6.2 and 7.6. The results showed that the tablets are completely dissolved within 15 minutes, and this indicates that the tablets can dissolve completely without any stress by chewing there are other. A machine is applied for chewable tablets, but using this one was starting to give a clear picture about the possible disintegration of tablets.

#### 2.4.2. Weight Variation

Tablets were randomly taken and weighed, and the mean weight was calculated. The percentage deviation of each chewing gum, from the mean, was calculated.

#### 2.4.3. Evaluation of Chewing Gums and Gummy Tablets (Gelatin)

Chewing gums and gummy tablets unlike tablets cannot be assayed by the conventional method through grinding the tablet and weighing an accurate amount of medicament and estimating its content. Therefore, the tablet has been dissolved and tested by TLC.

#### 2.4.4. TLC of Chewing Gum

The spot of meclizine and pyridoxine are applied on TLC and mobile phase used and compared to the results of spots displaced by new gelatin tablets that have been dissolved in water, and the same Rf was recorded for standard meclizine and pyridoxine to indicate the proper mixing of the active ingredients with other additives.

  Petroleum ether : acetone : methanol
  7 : 0.5 : 1

#### 2.4.5. Evaluation of Chewing Gum Consistency

The evaluation was carried out by the chew-out method. For evaluation of chewing gum consistency, the dummy chewing gums (without drug) were prepared according to the formula and then were given to the human volunteers to chew for a certain time.

#### 2.4.6. Assay Determination

The assay of the different tablet preparation was performed using HPLC, and the HPLC system used was a Beckman Gold system, prepared with a detector set at 254 nm, and a UV-vis spectrophotometer was used to evaluate the maximum absorption wavelength for both medicines. Standard solutions of approximately 50 mg pyridoxine hydrochloride and 25 mg meclizine hydrochloride were precisely weighed and dissolved in a 100 mL mobile phase. Sample solutions tablets were ground and mixed well. The amount is equivalent to 50 mg pyridoxine hydrochloride, and 25 mg meclizine hydrochloride was weighed and moved to a 100 mL volumetric flask. A mobile phase was added, and the solution was shaken for 15 min. The volume was made up to 100 mL with a mobile phase, and the solution was centrifugated. The column packed with a C18 silica gel, a flow rate of 1.5 mL/min, and a mobile phase (water, methanol, and acetonitrile (4 : 3 : 2)) was used. The resulting solutions were shaken for 30 min, and the volume was made up with the mobile phase. The concentration was determined in triplicate, and the % of the assay was determined.

### 2.5. In Vitro Study Release (Dissolution Test)

#### 2.5.1. Dissolution of Pyridoxine HCL and Meclizine

The drug release has been determined by measuring the concentration of the drug released after 15, 30, and 45 minutes in the medium of 0.01 M HCl in dissolution apparatus 2 with 100 rpm by withdrawing 5 ml of this solution in each time interval and diluted in a 50 ml volumetric flask.

*(1) Standard (STD) Solution*. 25 mg of meclizine HCl and 50 mg of pyridoxine HCL were taken into a 100 ml volumetric flask and dissolved in 10 ml methanol, then the solution was diluted with 0.01 M HCl. 5 ml of the solution was withdrawed and diluted in 50 ml volumetric flask. We put one tablet in each vessel and filter through a 0.45 *μ*g micromembrane filter.

*(2) Assay of Pyridoxine HCL and Meclizine*. Each tablet contains 25 mg meclizine HCl and 50 mg pyridoxine HCL anhydrous. *λ* = 230 nm. STD: prepared by dissolving in 100 ml HCl, then 10 ml was withdrawn to 50 ml flask and diluted with 0.1 M HCl. Sample (SP): crush the tablet and weigh an equivalent amount to 25 mg meclizine and 50 mg pyridoxine to 100 ml flask and dissolve in 10 ml methanol, then the volume is completed with 0.1 M HCl and withdrawn 10 ml to 50 ml volumetric flask and diluted with 0.1 M HCl.

*(3) Stationary Phase*. Silica gel. Mobile phase: 550 ml of 0.01 M HCl +350 ml acetonitrile +100 ml methanol.

*(4) Dissolution of Meclizine and Pyridoxine HCL Medium*. 900 ml of 0.07 M HCl, apparatus: 100 rpm. Time: 45 minutes. Std solution: 25 mg of meclizine HCl and 50 mg of pyridoxine HCL was weighed into 100 ml volumetric flask and diluted with 0.07 M HCl, then 5 ml of this solution was withdrawn and diluted in 50 ml volumetric flask. We put one tablet in each vessel and filter through a 0.45 *μ*g micromembrane filter.

## 3. Results and Discussion

Oral drug delivery is well known for many years because of the maximum broadly utilized routes of administration amongst all of the routes which have been employed for the systemic delivery of the drug via diverse pharmaceutical merchandise of different dosage paperwork. However, dysphagia (difficulty in swallowing) is common amongst all age corporations and greater, particularly with the pediatric, and geriatric populace not favorable, and might affect sufferers with nausea, vomiting, and motion illness headaches. Changing the form of meclizine and pyridoxine tablets into chewable tablets has been carried out in this research.

### 3.1. Survey

153 questionnaires were submitted, and the results showed that 88% of the respondents suffered from vomiting-associated pregnancy, and 49% from motion sickness. 58% of the respondents are pregnant women. 40% of responses are preferring chewable tablets, 46% for oral intake, and 8.6% for effervescent tablets. Meclizine and pyridoxine combination is the most usable dosage form by the response.

### 3.2. Formulation

Three types of formulations were prepared. The first preparation is gummy-based preparation and is most suitable for kids, and the other formulation is the gum-like and Mentos-like preparation.

#### 3.2.1. Chewing Gum-Like Formulation

Different base gum was used to prepare the gum-like formula using natural gums such as *Boswellia carterii* which gives hard and bitter formulations. Then, beeswax and acacia were used instead, but the formula was very soft. Because no pharm gum could be used in most gum preparation, we replace it by using prepared gum in the market that contains coloring, sucrose, and gum base. The color and sucrose were removed by washing them several times with distilled water, then melted, and mixed with other excipients. The citric acids were added to mask the bitter taste of pyridoxine and in a combination of sucrose to give the sweet taste. Then, the estimation of chewing gum consistency is determined and summarized in Table 1.

*(1) Chewable Tablet: Mentos-Like Formulation*. Four trials were carried out to get the most accepted formulation by volunteers and as consistency where the first three formulas were modified because of sour taste or the high elasticity that has been corrected by controlling the number of citric acid amounts, glycerin, and xanthan gum (Table 2).

*(2) Gummy-Like Preparation*. Seven formulations were prepared and failed because of different reasons such as contamination, sour preparation, and other problem. Different formulations were prepared because of the problem of contamination as gelatin is a good source of microbial contamination and is overcome by adding preservatives. Also, the softening of the formulation was changed till the right amount was carried out.

Ten volunteers tested the consistency of the tablets, and most of them liked the preparation, except for two of them who had comments about the bitter and sour tastes; therefore, the formulation was approved and considered accepted, as displayed in Table 3. Also, these results support the results of a survey that prefers chewable tablets to oral tablets to relieve nausea and vomiting.

*(3) Preidentification by TLC of Chewing Gum*

  Rf = *a*/*b*
  Standard of meclizine (*a*) = 2.
  Standard of pyridoxine (*a*) = 2.3 *b* = 7.2
  Sample of meclizine in gum tablet (*a*) = 1.8
  Sample of pyridoxine in gum tablet (*a*) = 2.3
  Rf = *a*/*b*
  Rf of meclizine (standard) = 2/7.2 = 0.27
  Rf of pyridoxine (standard) = 2.3/7.2 = 0.3
  Rf of meclizine in gum tablet (sample) = 1.8/7.2 = 0.25
  Rf of pyridoxine in gum tablet (sample) = 2.3/7.2 = 0.3

By comparing the result of pure meclizine and pyridoxine with the new gum formulation, it is shown that the formula gives the same spot at the same level of pyridoxine Rf (0.3) and meclizine Rf (0.25) compared to a standard drug that gives Rf (0.3) pyridoxine Rf (0.27) meclizine. This indicated that the formula was mixed and prepared correctly and was confirmed by giving the same Rf.

*(4) Preidentification by TLC of Gelatin Tablet*

  Rf = *a*/*b*
  Standard of meclizine (*a*) = 3.8
  Standard of pyridoxine (*a*) = 3.2 (*b*) = 6.9
  Sample of meclizine in gelatin tablet (*a*) = 3.9 and sample of pyridoxine in gelatin tablet (*a*) = 3.3
  Rf = *a*/*b*
  Rf of meclizine (standard) = 3.8/6.9 = 0.55
  Rf of pyridoxine (standard) = 3.2/6.9 = 0.4
  Rf of meclizine in gelatin tablet (sample) = 3.9/6.9 = 0.56
  Rf of pyridoxine in gelatin table (sample) *t* = 3.3/6.9 = 0.47

By comparing the result of pure meclizine and pyridoxine with our formulation, it is shown that our formula gives the same spot at the same level of pyridoxine Rf (0.47) and meclizine Rf (0.65) compared to standard drug that gives Rf (0.4) pyridoxine and Rf (0.55) meclizine. This indicates that the formula was mixed, prepared correctly, and mixed within all tablets, and all samples showed same RF and spot size.

### 3.3. Weight Variation

Tablet weight variation as displayed in Table 4 showed that chewable tablets indicate that the molds and process carried out were accurate and filled within the limit.

### 3.4. Assay Determination

The different tablet formulations were determined by HPLC and the assay of gum-like tablets, Mentos-like tablets, and gummy-like tablets gives the % of assay 98% ± 0.05, 99.3 ± 0.08, and 98.7 ± 0.4, respectively, for meclizine and 99.2 ± 0.02, 97 ± 0.09, and 100.9 ± 0.08 for pyridoxine. This is to ensure the formulations are well prepared (Table 5).

## 4. Dissolution Result

The result showed the dissolution profile for the three prepared formulations after different time intervals of 15, 30, and 45 min as summarized in Table 5 to indicate the proper release of the prepared formulation.

## 5. Conclusion

Three types of chewable tablet formulations of meclizine and pyridoxine are prepared and give promising properties and are acceptable to the volunteers used.

## Figures and Tables

**Table 1:** Estimation of chewing gum consistency for chewable (gum-like) tablets.

Volunteers	Sex	Age	Observation
1	Male	18	Good
2	Male	25	Good
3	Male	27	Good
4	Male	25	Good
5	Male	25	Good
6	Male	11	Good
7	Female	25	Good
8	Female	25	Good
9	Female	10	Good
10	Male	25	Good
11	Male	20	Good
12	Male	25	Good
13	Male	25	Good
14	Male	20	Good
15	Male	13	Good
16	Female	10	Acceptable
17	Female	35	Acceptable
18	Female	40	Good

**Table 2:** Estimation of chewing gum consistency for chewable (Mentos-like) tablets.

No	Age	Sex	Comment
1	21	Female	Good taste and sweet
2	26	Male	Good taste, sweet taste with bitter taste
3	20	Male	Good taste
4	8	Male	Good taste
5	10	Male	Good taste
6	7	Female	Good taste and sweat
7	9	Male	Sweat taste
8	13	Female	Sour taste
9	15	Female	Bitter taste
10	11	Female	Good taste

**Table 3:** Estimation of chewing gum consistency for gummy (gelatin) tablets.

Volunteers	Sex	Age	Observation
1	Male	18	Good
2	Male	25	Good
3	Male	27	Good
4	Male	25	Good
5	Male	25	Good
6	Male	11	Good
7	Female	25	Good
8	Female	25	Good
9	Female	10	Acceptable
10	Male	25	Good
11	Male	20	Good
12	Male	25	Good
13	Male	25	Good
14	Male	20	Good
15	Male	13	Good
16	Female	10	Acceptable
17	Female	25	Good
18	Female	40	Good

**Table 4:** Weight variation of the chewable formulation.

No of tablets	Weights (g) gum-like tablet	Weights (g) Mentos-like tablet	Weights (g) gelatin-like tablet
1	0.92	1.03	1.92
2	1.03	1.01	2.0
3	0.98	0.99	1.8
4	0.96	0.93	1.97
5	1	0.99	1.92
6	0.98	0.98	1.99
7	0.99	0.99	2
8	0.97	1	1.98
9	1	0.97	1.92
10	1	1.02	1.95
Average weight: 0.984 g		0.991 g	1.95 g
Limit: (1.03−0.94) g		(1.04−0.94) g	(2.037−1.8) g

**Table 5:** Dissolution profile and assay for the prepared formulation.

	Meclizine	Pyridoxine	Meclizine	Pyridoxine	Meclizine	Pyridoxine
	*Gum-like tablet*	*Mentos-like tablet*			*Gelatin-like tablet*	
15_min_	78.7%	51.12%	88.66%	63.22%	72.16%	97.13%
30_min_	92.45%	90.06%	90.12%	100.34%	102.19%	105.14%
45_min_	97.59%	99.25%	95.53%	100.8%	102.8%	115%
Assay	98% ± 0.05	99.2 ± 0.02	99.3 ± 0.08	100.9 ± 0.08	98.7 ± 0.4	97 ± 0.09

## Data Availability

The data can be provided upon request from the corresponding author.
